# Availability of Investigational Medicines Through the US Food and Drug Administration’s Expanded Access and Compassionate Use Programs

**DOI:** 10.1001/jamanetworkopen.2018.0283

**Published:** 2018-06-15

**Authors:** Jeremy Puthumana, Jennifer E. Miller, Jeanie Kim, Joseph S. Ross

**Affiliations:** 1 Yale School of Medicine, New Haven, Connecticut; 2 Division of Medical Ethics, Department of Population Health, New York University School of Medicine, Bioethics International, New York; 3 Collaboration for Research Integrity and Transparency (CRIT), Yale Law School, New Haven, Connecticut; 4 Section of General Medicine, Department of Internal Medicine, Yale School of Medicine, New Haven, Connecticut; 5 Department of Health Policy and Management, Yale School of Public Health, New Haven, Connecticut; 6 Center for Outcomes Research and Evaluation, Yale–New Haven Hospital, New Haven, Connecticut

## Abstract

**Importance:**

The Right to Try Act of 2017 allows patients with life-threatening conditions to access investigational medicines outside clinical trials without oversight from the US Food and Drug Administration (FDA). A better understanding of existing expanded access programs can inform the consideration and implementation of both the federal Right to Try Act and state right-to-try laws.

**Objective:**

To determine the timing and duration of expanded access programs for investigational medicines initiated prior to FDA approval.

**Design and Setting:**

This cross-sectional study examined expanded access and compassionate use programs registered through August 1, 2017, identified from ClinicalTrials.gov and publicly available FDA documents.

**Main Outcomes and Measures:**

Start date of each program and 3 key regulatory dates (investigational new drug application activation, initial new drug application submission, and FDA approval), and timing and duration of expanded access availability in relation to new drug application submission and FDA approval.

**Results:**

Through ClinicalTrials.gov, 92 FDA-approved drugs and biologics with associated expanded access programs initiated prior to FDA approval were identified. These programs were initiated between September 1996 and June 2017 for medicines that were most commonly used to treat cancer (n = 46 [50.0%]); metabolic, endocrine, and genetic diseases (n = 16 [17.4%]); and infectious diseases (n = 14 [15.2%]). The median (interquartile range) premarket expanded access availability was 10.0 (6.0-19.5) months, constituting a median (interquartile range) of 14% (7%-25%) of the premarket clinical development period (investigational new drug application activation to FDA approval). Of 92 expanded access programs, 64 (69.6%) were initiated just before or after new drug application submission: 24 (26.1%) were initiated during the 6-month period before, and 40 (43.5%) in the 6 months after.

**Conclusions and Relevance:**

Over the past 2 decades, expanded access programs have provided access to investigational medicines for which evidence of safety and effectiveness was established. For medicines that ultimately receive FDA approval, these findings suggest that the FDA and pharmaceutical industry have established a balance between investigational new drug access and protection of patients from therapies without established safety. This balance may be compromised by policy makers seeking to speed access to investigational medicines through the Right to Try Act.

## Key Points

**Question:**

What is the timing and duration of investigational drug availability through the US Food and Drug Administration’s (FDA’s) expanded access program?

**Findings:**

In this cross-sectional study of 92 FDA-approved drugs with associated expanded access programs, the median premarket expanded access availability was 10 months. Of 92 expanded access programs, 64 (69.6%) were initiated within the 6 months preceding or following a new drug’s application submission to the FDA.

**Meaning:**

The FDA and the pharmaceutical industry have established a balance between investigational new drug access and patient safety that may be compromised by policy makers seeking to speed access to investigational medicines through the Right to Try Act.

## Teaser

This study describes expanded access and compassionate use programs created through 2017 for experimental drugs and biologics, and the timing and duration of existence of those programs relative to the date of US Food and Drug Administration (FDA) final approval.

## Introduction

The Right to Try Act of 2017, recently passed by the Senate and under consideration by the House of Representatives, allows patients with life-threatening conditions to obtain investigational medicines without oversight and approval from the US Food and Drug Administration (FDA).^1^ As of March 2018, right-to-try legislation has been enacted in 38 states. Advocates of the federal Right to Try Act, which was strongly endorsed by President Donald J. Trump in his 2018 State of the Union Address, contend that it provides potentially life-saving treatments to patients who have exhausted all approved therapies and who do not qualify for or have access to clinical trials. Skeptics argue that patients can already gain access to investigational medicines through the FDA’s existing expanded access program, which grants more than 99% of the patient requests received for investigational medicines. Access to the investigational agent is then contingent on the manufacturer providing the drug. For emergency single-patient requests, the agency typically responds within hours.^2^

The FDA’s expanded access program was originally designed to provide access to investigational medicines for which there is sufficient clinical evidence of safety and effectiveness with authorization from the commercial sponsor (commonly the pharmaceutical industry).^3,4^ Thus, the FDA is balancing 2 competing priorities: investigational new drug access and protection of patients from therapies without established safety. To our knowledge, no studies have examined the timing and duration of drugs made available through expanded access programs to determine whether the program was serving its original purpose. To inform consideration of the Right to Try Act, we used ClinicalTrials.gov to identify investigational medicines made available through expanded access programs prior to FDA approval and determined for what proportion of the premarket clinical development period manufacturers maintained these programs.

## Methods

We searched ClinicalTrials.gov for all expanded access and compassionate use programs registered through August 1, 2017. Although no formal start date was applied to the search, the first expanded access program registered on ClinicalTrials.gov was initiated in September 1996. We did not limit the search. Keywords used for the search included *expanded access* and *compassionate use*. We manually reviewed the search results for relevance and removed duplicate trials (trials with the same ClinicalTrials.gov identifier). For each identified program, we abstracted expanded access program location, start date, and therapeutic intervention.^5^ We contacted the corresponding program sponsor to request these data if they were not available on ClinicalTrials.gov; every program sponsor that was contacted provided the requested information. Next, we used the Drugs@FDA database to determine whether the investigational medicine ultimately received FDA approval, conducting our search in August 2017. We limited our sample to investigational drugs and biologics that were made available through expanded access in the United States prior to FDA approval. When multiple programs were available for the same medicine, we included the earliest.

For each medicine, we identified 3 regulatory dates using the Drugs@FDA database: investigational new drug (IND) application activation, initial new drug application (NDA) submission to the FDA, and FDA approval of the NDA. If the IND application activation date was not available from Drugs@FDA, the information was abstracted from FDA documents, administrative correspondence, and patent extension notices. Using these dates, we determined the total clinical development period (IND activation to FDA approval of the NDA), the clinical research period (IND activation to NDA submission), the FDA review period (NDA submission to FDA approval of the NDA), and time the medicines were available through expanded access (expanded access program initiation to FDA approval of the NDA). We then calculated expanded access availability by dividing the time medicines were available through expanded access by the total clinical development period.

We used data from Drugs@FDA to determine the following therapeutic and regulatory characteristics for each investigational medicine that ultimately received FDA approval: agent type (drug or biologic), orphan status, fast-track designation, breakthrough therapy designation (a designation established in 2012 for therapies that demonstrate substantial improvement over available therapy), accelerated approval, priority review designation, and black box warning at the time of FDA approval. For each investigational medicine, we also determined its primary therapeutic area and whether it was used to treat a life-threatening condition.

We used Wilcoxon and Kruskal-Wallis tests as appropriate to examine differences in expanded access availability across therapeutic and regulatory characteristics. We used Wilcoxon, Fisher exact, and χ^2^ tests as appropriate to examine differences in therapeutic and regulatory characteristics by timing of expanded access program initiation. Analyses were conducted using Excel version 14.1.0 (Microsoft Corp) and Stata statistical software version 13.0 (StataCorp LLC). All statistical tests were 2-sided and accounted for multiple comparisons (n = 10) by using a *P* value of .005 for statistical significance for all subgroup analyses.

This cross-sectional study was prepared in accordance with the Strengthening the Reporting of Observational Studies in Epidemiology (STROBE) reporting guideline. This study did not require institutional review board approval or patient informed consent because it is based on publicly available data and involved no patient records.

## Results

There were 92 FDA-approved drugs and biologics with associated expanded access programs initiated prior to FDA approval identified from ClinicalTrials.gov; 90 (97.8%) received FDA approval for the expanded access indication. These programs were initiated between September 1996 and June 2017. The most common therapeutic uses were to treat cancer (n = 46 [50.0%]); metabolic, endocrine, and genetic diseases (n = 16 [17.4%]); and infectious diseases (n = 14 [15.2%]) (Table 1). The FDA granted orphan status to 59 medicines (64.1%), and of the 39 NDAs filed after the breakthrough designation was created, 23 medicines (59.0%) were so designated.

**Table 1.** Review Periods for Investigational Medicines With Expanded Access Programs Prior to Regulatory Approval

Medicine Characteristic	Medicines, No. (%)	Duration, Median (IQR), mo				EAP Proportion, Median (IQR), %^a^	*P* Value
		IND Activation to FDA Approval	IND Activation to NDA Submission	NDA Submission to FDA Approval	EAP Initiation to FDA Approval		
Overall	92 (100.0)	77.5 (60.0-116.5)	69.0 (52.5-107.0)	7.5 (6.0-11.0)	10.0 (6.0-19.5)	14 (7-25)	
Therapeutic area							
Cancer	46 (50.0)	86.5 (62.0-125.0)	80.5 (57.0-112.0)	6.0 (5.0-9.0)	9.0 (6.0-16.0)	12 (5-21)	.06
Metabolic, endocrine, or genetic	16 (17.4)	73.0 (42.5-84.5)	60.0 (35.5-73.5)	10.0 (6.5-11.5)	9.0 (3.0-15.0)	14 (5-29)	
Infectious disease	14 (15.2)	62.0 (48.0-92.0)	52.0 (40.0-76.0)	6.0 (6.0-8.0)	13.5 (8.0-22.0)	22 (18-36)	
Other	16 (17.4)	109.0 (70.0-148.0)	91.0 (63.5-126.0)	10.0 (6.5-18.0)	9.5 (5.5-35.0)	10 (6-20)	
Agent type							
Drug	63 (68.5)	73.0 (57.0-115.0)	66.0 (53.0-103.0)	6.0 (5.0-9.0)	10.0 (6.0-16.0)	14 (6-25)	.97
Biologic	29 (31.5)	96.0 (61.0-125.0)	92.0 (52.0-110.0)	9.0 (6.0-12.0)	10.0 (6.0-29.0)	14 (7-25)	
Orphan status							
Yes	59 (64.1)	84.0 (60.0-125.0)	74.0 (53.0-112.0)	8.0 (6.0-11.0)	10.0 (5.0-28.0)	14 (6-25)	.60
No	33 (35.9)	68.0 (60.0-102.0)	61.0 (51.0-94.0)	6.0 (6.0-9.0)	10.0 (6.0-16.0)	18 (8-25)	
Fast track							
Yes	60 (65.2)	78.5 (60.5-114.0)	69.0 (54.5-102.0)	8.0 (5.0-10.5)	10.0 (6.0-22.5)	14 (6-29)	.68
No	31 (33.7)	79.0 (60.0-128.0)	71.0 (50.0-120.0)	7.0 (6.0-11.0)	9.0 (6.0-16.0)	14 (7-24)	
Not applicable^b^	1 (1.1)	20.0 (20.0-20.0)	17.0 (17.0-17.0)	3.0 (3.0-3.0)	6.0 (6.0-6.0)	30 (30-30)	
Breakthrough therapy							
Yes	23 (25.0)	69.0 (48.0-105.0)	61.0 (40.0-96.0)	7.0 (5.0-10.0)	6.0 (3.0-10.0)	7 (4-17)	.97
No	16 (17.4)	116.5 (73.5-170.0)	108.5 (66.5-160.0)	11.0 (7.5-12.0)	10.0 (3.5-14.0)	10 (3-16)	
Not applicable^b^	53 (57.6)	74.0 (61.0-113.0)	67.0 (54.0-103.0)	6.0 (6.0-10.0)	14.0 (7.0-29.0)	18 (9-35)	
Priority review							
Yes	76 (82.6)	73.5 (56.5-113.0)	67.5 (50.5-102.0)	6.0 (5.0-8.5)	8.0 (5.5-16.0)	14 (7-24)	.49
No	16 (17.4)	108.5 (76.0-152.0)	97.0 (55.5-124.0)	13.0 (11.0-25.5)	15.0 (10.5-31.0)	16 (7-38)	
Accelerated approval							
Yes	28 (30.4)	62.5 (47.0-98.5)	56.5 (39.5-94.0)	6.0 (5.0-9.5)	9.0 (6.0-16.0)	17 (9-29)	.25
No	64 (69.6)	87.5 (68.0-126.5)	77.5 (58.5-115.0)	8.0 (6.0-11.0)	10.0 (5.0-22.0)	12 (6-22)	
Black box warning							
Yes	29 (31.5)	74.0 (62.0-115.0)	68.0 (57.0-107.0)	8.0 (6.0-11.0)	9.0 (6.0-22.0)	14 (7-29)	.98
No	63 (68.5)	81.0 (59.0-118.0)	70.0 (52.0-107.0)	7.0 (5.0-11.0)	10.0 (6.0-16.0)	14 (6-24)	
Life-threatening condition							
Yes	82 (89.1)	74.0 (59.0-115.0)	68.0 (52.0-107.0)	7.0 (5.0-10.0)	10.0 (6.0-18.0)	14 (6-25)	.97
No	10 (10.9)	104.5 (73.0-149.0)	92.5 (57.0-124.0)	10.0 (7.0-25.0)	11.0 (6.0-23.0)	13 (8-20)	
Year of approval							
Prior to 2011	29 (31.5)	68.0 (50.0-98.0)	58.0 (39.0-92.0)	6.0 (6.0-9.0)	13.0 (6.0-22.0)	18 (9-36)	.01
2011-2014	36 (39.1)	83.0 (62.5-115.0)	72.5 (58.0-105.5)	8.0 (5.0-10.0)	10.5 (6.0-30.5)	14 (7-25)	
After 2014	27 (29.4)	96.0 (64.0-130.0)	92.0 (56.0-124.0)	8.0 (5.0-11.0)	8.0 (4.0-11.0)	8 (3-17)	

Abbreviations: EAP, expanded access program; FDA, US Food and Drug Administration; IND, investigational new drug; IQR, interquartile range; NDA, new drug application.

^a^ The EAP proportion is the length of time an agent was available through expanded access (program initiation to FDA approval) divided by the premarket clinical development period (IND to FDA approval).

^b^ Comparisons excluded for medicines whose review took place before the fast-track and breakthrough therapy designations existed.

The median (interquartile range [IQR]) premarket clinical development period (IND activation to FDA approval) was 77.5 (60.0-116.5) months. Of this time, the median (IQR) clinical research period (IND activation to NDA submission) was 69.0 (52.5-107.0) months, while the median (IQR) FDA review period (NDA submission to approval) was 7.5 (6.0-11.0) months. Median (IQR) premarket expanded access availability (program initiation to FDA approval) was 10.0 (6.0-19.5) months, constituting a median (IQR) of 14% (7%-25%) of the premarket clinical development period.

There were no significant differences in expanded access availability across all other therapeutic and regulatory characteristics (Table 1). For instance, the proportion of the premarket clinical development period for which there was expanded access availability was not statistically different for medicines used to treat cancer (median [IQR], 12% [5%-21%]), metabolic, endocrine, and genetic diseases (median [IQR], 14% [5%-29%]), or infectious diseases (median [IQR], 22% [18%-36%]) or for treatments for other conditions (median [IQR], 10% [6%-20%]) (*P* = .06). In addition, the proportion of expanded access availability was no different for drugs receiving the fast-track approval designation (median [IQR], 14% [6%-29%] vs 14% [7%-24%]; *P* = .68) and the accelerated approval designation (median [IQR], 17% [9%-29%] vs 12% [6%-22%]; *P* = .25), which are granted to drugs that treat serious conditions and fill an unmet medical need. There was also no difference for drugs receiving the breakthrough therapy designation (median [IQR], 7% [4%-17%] vs 10% [3%-16%]; *P* = .97).

Of 92 expanded access programs, 64 (69.6%) were initiated just before or after NDA submission: 24 (26.1%) were initiated during the 6-month period prior and 40 (43.5%) in the 6 months after (Figure). There were no significant differences in the timing of expanded access program initiation (>6 months prior to NDA submission vs ≤6 months prior to NDA submission) by therapeutic and regulatory characteristics (Table 2). For instance, the proportion of investigational medicines used to treat cancer (14 of 28 [50.0%] vs 32 of 64 [50.0%]), metabolic, endocrine, and genetic diseases (3 of 28 [10.7%] vs 13 of 64 [20.3%]), or infectious disease (6 of 28 [21.4%] vs 8 of 64 [12.5%]) or for treatment for other conditions (5 of 28 [17.9%] vs 11 of 64 [17.2%]) was not statistically different by timing of expanded access program initiation (*P* = .58). Notably, there were also no significant differences in the duration of the clinical development periods (>6 months prior to NDA submission, median [IQR], 89.5 [59.5-138.0] months vs ≤6 months prior to NDA submission, median [IQR], 74.0 [60.0-113.0] months; *P* = .22), clinical research periods (>6 months prior to NDA submission, median [IQR], 83.5 [53.5-129.0] months vs ≤6 months prior to NDA submission, median [IQR], 67.5 [52.5-101.5] months; *P* = .23), or FDA review periods (>6 months prior to NDA submission, median [IQR], 6.0 [5.5-11.0] months vs ≤6 months prior to NDA submission, median [IQR], 8.0 [6.0-10.5] months; *P* = .89) based on the timing of expanded access program initiation.

**Figure.**
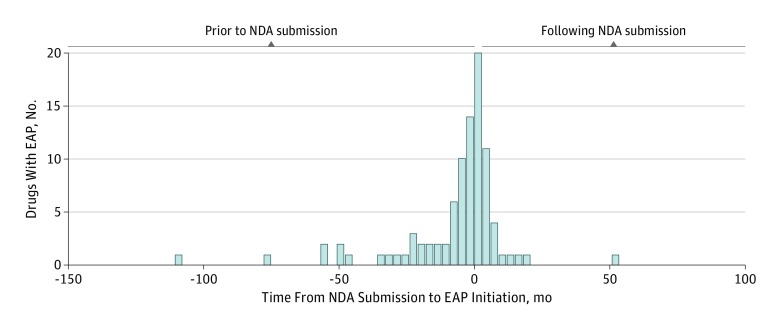
Distribution of Investigational Medicine by Time of New Drug Application (NDA) Submission to Expanded Access Program (EAP) Initiation Of 92 expanded access programs, 64 (69.6%) were initiated just before or after NDA submission: 24 (26.1%) were initiated during the 6 months before and 40 (43.5%) in the 6 months after.

**Table 2.** Comparison of Therapeutic and Regulatory Characteristics for Investigational Medicines

Medicine Characteristic	Medicines, No. (%)	EAP Initiation >6 mo Before NDA Submission, No. (%)	EAP Initiation ≤6 mo Before NDA Submission, No. (%)	*P* Value
Overall	92 (100)	28 (30.4)	64 (69.6)	
Therapeutic area				
Cancer	46 (50.0)	14 (50.0)	32 (50.0)	.58
Metabolic, endocrine, or genetic	16 (17.4)	3 (10.7)	13 (20.3)	
Infectious disease	14 (15.2)	6 (21.4)	8 (12.5)	
Other	16 (17.4)	5 (17.9)	11 (17.2)	
Agent type				
Drug	63 (68.5)	19 (67.9)	44 (68.8)	.93
Biologic	29 (31.5)	9 (32.1)	20 (31.3)	
Orphan status				
Yes	59 (64.1)	16 (57.1)	43 (67.2)	.36
No	33 (35.9)	12 (42.9)	21 (32.8)	
Fast track				
Yes	60 (65.2)	19 (67.9)	41 (64.1)	.80
No	31 (33.7)	9 (32.1)	22 (34.4)	
Not applicable^a^	1 (1.1)	0	1 (1.6)	
Breakthrough therapy				
Yes	23 (25.0)	1 (3.6)	22 (34.4)	.29
No	16 (17.4)	3 (10.7)	13 (20.3)	
Not applicable^a^	53 (57.6)	24 (85.7)	29 (45.3)	
Priority review				
Yes	76 (82.6)	23 (82.1)	53 (82.8)	.94
No	16 (17.4)	5 (17.9)	11 (17.2)	
Accelerated approval				
Yes	28 (30.4)	9 (32.1)	19 (29.7)	.81
No	64 (69.6)	19 (67.9)	45 (70.3)	
Black box warning				
Yes	29 (31.5)	10 (35.7)	19 (29.7)	.57
No	63 (68.5)	18 (64.3)	45 (70.3)	
Life-threatening condition				
Yes	82 (89.1)	25 (89.3)	57 (89.1)	>.99
No	10 (10.9)	3 (10.7)	7 (10.9)	
Year of approval				
Prior to 2011	29 (31.5)	13 (46.4)	16 (25.0)	.02
2011 to 2014	36 (39.1)	12 (42.9)	24 (37.5)	
After 2014	27 (29.4)	3 (10.7)	24 (37.5)	
Regulatory review period, median (IQR), mo				
IND activation to FDA approval	92 (100)	89.5 (59.5-138.0)	74.0 (60.0-113.0)	.22
IND activation to NDA submission	92 (100)	83.5 (53.5-129.0)	67.5 (52.5-101.5)	.23
NDA submission to FDA approval	92 (100)	6.0 (5.5-11.0)	8.0 (6.0-10.5)	.89

Abbreviations: EAP, expanded access program; FDA, US Food and Drug Administration; IND, investigational new drug; IQR, interquartile range; NDA, new drug application.

^a^ Comparisons excluded for medicines whose review took place before the fast-track and breakthrough therapy designations existed.

## Discussion

Of 92 expanded access programs registered on ClinicalTrials.gov over the past 2 decades for which investigational medicines ultimately received FDA approval, 64 (69.6%) were initiated within the 6 months preceding or following NDA submission. Overall, these medicines were made available to patients for 14% of their clinical development period, and there were no differences in expanded access availability across therapeutic and regulatory characteristics. To our knowledge, this is the first study to evaluate the timing and duration of drug availability through expanded access programs, and our findings can be used to inform ongoing discussions over the framework and final details of the federal Right to Try Act and state right-to-try laws.

Consistent with the original intent of expanded access programs, which is to provide access to investigational medicines with sufficient evidence of safety and/or effectiveness,^3^ our findings suggest these medicines are generally made available after clinical research needed for FDA approval is completed or is nearing completion—around the time of NDA submission. Thus, for medicines that ultimately receive FDA approval, expanded access programs over the past 2 decades are providing access to investigational medicines for which safety and effectiveness have been established. These findings suggest the FDA and the pharmaceutical industry have established a balance between investigational new drug access and protection of patients from therapies without established safety, which may be compromised by policy makers seeking to speed access to investigational medicines through the Right to Try Act by removing the requirement for FDA oversight and approval of expanded access requests.

### Limitations

Our study was limited to programs registered on ClinicalTrials.gov and does not account for programs or individual patient requests to manufacturers that were not registered. Expanded access program registration requirements were only recently clarified under the final rule^6^; prior to this recent clarification, the FDA did not enforce the registration requirement. In addition, we could not assess the proportion of post–phase 1 period drugs made accessible through expanded access because no reliable data were available specifying the end date of phase 1 development. In addition, our analysis was limited to investigational medicines that ultimately received FDA approval; fewer than half of such medicines are ever approved by the FDA,^7^ potentially limiting generalizability but reinforcing the importance of requiring sufficient evidence of safety and/or efficacy prior to expanded access. Finally, for clarity, the sample used for this study differs from that used in our prior work^5^; the current study was intended to better inform the pending federal right-to-try legislation by including only expanded access programs that offered drug availability in the United States and by including only the earliest expanded access programs identified for those therapeutics for which multiple programs were available.

## Conclusions

Our results suggest that the FDA’s existing expanded access program serves to provide access to investigational medicines once sufficient evidence of safety and efficacy has been generated by sponsors, a balance intended to serve the best interests of patients. The Right to Try Act, on the other hand, encourages sponsors to make investigational medicines available earlier in the clinical development period, in part because it shields them from any liability associated with patient injury due to expanded access.^4^ Nevertheless, under both the FDA’s expanded access program and the proposed Right to Try Act, sponsors may still refuse access to their investigational medicines. Therefore, legislative efforts aiming to safely ensure drug availability should work to involve both investigational medicine manufacturers and the FDA. Such legislation may help patients with life-threatening conditions gain access to investigational medicines without compromising patient safety or the process for evaluating drug efficacy and safety established by the FDA.
